# The Impact of Drug-induced Sleep Endoscopy on Therapeutic Decisions in Obstructive Sleep Apnea: A Systematic Review and Meta-analysis

**DOI:** 10.7759/cureus.6041

**Published:** 2019-10-30

**Authors:** Abdullah A Albdah, Meshael M Alkusayer, Mohammed Al-Kadi, Hesham Almofada, Ebraheem A Alnofal, Sara Almutairi

**Affiliations:** 1 Otolaryngology, King Salman bin Abdulaziz Hospital, Riyadh, SAU; 2 Otorhinolaryngology - Head and Neck Surgery, King Saud Medical City, Riyadh, SAU; 3 Otolaryngology - Head & Neck Surgery, King Abdulaziz Medical City, Riyadh, SAU; 4 Otolaryngology - Head & Neck Surgery, King Faisal Specialist Hospital & Research Centre, Riyadh, SAU; 5 Otorhinolaryngology - Head & Neck Surgery, King Saud Medical City, Riyadh, SAU

**Keywords:** obstructive sleep apnea, sleep disorder, drug-induced sleep endoscopy

## Abstract

Objective

The aim of this study was to assess the ability of drug-induced sleep endoscopy (DISE) to change therapeutic decisions through the identification of obstruction sites in patients with obstructive sleep apnea (OSA).

Materials and methods

A systematic review and meta-analysis were conducted concerning studies that reported the impact of DISE on therapeutic recommendations. The percentage of change was collected for each study and per site of the collapse. The pooled rate of change and the respective 95% confidence interval (CI) were computed. Subgroup analysis was performed based on patients’ age, sample size, the applied DISE protocol, and the originally used diagnostic modality before DISE.

Results

In a total of nine studies, 1247 patients were included (69.2% males, 59.7% children, 78.04% with a multilevel collapse). Therapeutic decisions changed in 43.69% of patients (CI, 33.84 to 53.54). The change rates were significantly higher in adults (54.0% versus 25.9% in children, P = 0.001), midazolam-based DISE protocols (78.4% versus 48.45% for midazolam plus propofol and 33.9% for propofol, *P *< 0.001), and after awake endoscopy (62.2% as compared to 44.6% after clinical basic examination [CBE], 40.1% after CBE, lateral cephalometry, and Müller maneuver, *P *= 0.02). Changes at uvular and palatal sites were more frequent in adults and at the tonsils in children.

Conclusion

The DISE approach can be promoted via implementing unified classification systems of obstruction sites; the widescale application of target-controlled infusion and its therapeutic benefits can be explored in well-designed randomized studies that compare its efficacy with other diagnostic modalities.

## Introduction

Obstructive sleep apnea (OSA) is a sleep disorder characterized by multiple episodes of partial or complete obstruction of the upper airway, which leads to sympathetic activation, brain arousal, and changes in oxygen saturation in the blood [1]. The pharynx constitutes the most common sites of obstruction, such as soft palate elongation, swollen uvula, a large tongue, large tonsils, and thickened pharyngeal mucosa. These changes are consequently associated with insomnia, exaggerated day-time sleepiness, and tiredness, yet some patients may be asymptomatic. Additionally, OSA may be a risk factor of endocrinologic abnormalities, cardiovascular diseases, and mortality [2].

The gold standard of OSA diagnosis is a polysomnogram (PSG), which relies on calculating the number of airway obstructive events per hour of sleep, and is named the apnea-hypopnea index (AHI). A confirmed diagnosis is deemed at an AHI ≥ 5, and disease severity depends on the frequency of events. Considering these diagnostic criteria, OSA can be prevalent among 9% to 38% of the general population and it is higher among men, elderly people, and increased body mass index (BMI) [3]. Therefore, intuitively, OSA prevalence has substantially increased in concordance with the trend of obesity prevalence over the past 25 years [4].

Continuous positive airway pressure (CPAP) is the mainstay nonsurgical treatment approach in patients with moderate to severe OSA, but poor compliance limits its feasibility [5]. Therefore, several surgical modalities target the airway structures contributing to obstruction. For instance, surgeries comprising the removal of soft tissue structures include adenoidectomy, tonsillectomy, uvulopalatopharyngoplasty (UPPP), midline glossectomy, and radiofrequency ablation of the palate (RFP), base of the tongue (RFBT) or turbinates. Furthermore, modifications of the skeletal/soft tissues can be performed, such as septoplasty, genioglossal advancement, and mandibular or maxillomandibular advancement [6].

The outcomes of the treatment approaches are essentially dependent on the definitive identification of obstruction sites and the perfect selection of eligible patients. As such, it is important to utilize reliable methods of upper airway evaluation. Of these methods, drug-induced sleep endoscopy (DISE) has proven effective to predict snoring/OSA based on partial airway obstruction findings via direct observation of the obstructed site(s) [7]. The technique was first described three decades ago at the Nose and Ear Hospital in the United Kingdom as an alternative to a pre-established time-consuming technique, which comprises endoscopic evaluation during natural sleep. In DISE procedures, propofol, midazolam, or dexmedetomidine are administered in single or combined doses to attain a specific depth of sedation (loss of response to verbal communication). In such methods, target-controlled infusion (TCI) of sedative agents has the potential to decrease the chances of profound muscular relaxation and thus reduce the false-positive reporting of obstructive events [8]. Hence, DISE is a safe procedure in the hands of an experienced anesthesiologist.

However, the ability of DISE to detect obstructive abnormalities as compared to other evaluative approaches, such as clinical basic examination (CBE) by ear, nose and throat clinicians, is rarely investigated. This discriminative ability might eventually change the initially proposed management plan and could have significant effects on the patient’s outcomes. Hence, we sought to analytically review the available evidence regarding the rates of changed managemental indications with DISE use as compared to other diagnostic modalities. Moreover, the outcomes of DISE-directed approaches were matched to those obtained by other methods.

## Materials and methods

Search strategy and study selection

The main outline and strategy of the present study were implemented according to the guidelines of the Preferred Reporting Items for Systematic Reviews and Meta-analyses (PRISMA). The following databases were thoroughly searched for eligible studies: PubMed, Web of Science, Embase, and Google Scholar. The search process was performed for studies published between 2000 and 2019. The last access to all databases was on August 18, 2019. The reference lists of the screened articles were additionally searched for eligible articles. Two authors independently carried out the search process and any disagreement was resolved by discussion.

Primary and secondary outcomes

The frequency of patients whose initially established OSA therapy was changed after undergoing DISE was considered the primary outcome. Secondary outcomes included the collapsed sites that were best detected by DISE. In addition, changes in the sleep parameters, such as the AHI and the Epworth sleepiness scale (ESS), after treatment is driven by both modalities, were collected whenever possible. 

Eligibility criteria

Retrospective analyses and prospective cohort studies reporting the potential of DISE use on changing the therapeutic decisions in OSA patients were eligible. All diagnostically confirmed cases with OSA of all ages were included. The minimum sample size was 25 patients. The diagnostic modality used in making the initial therapeutic decision as well as medications used for sedation in DISE was explicitly mentioned. Studies providing insufficient data regarding the primary outcomes were excluded. Besides, non-English articles, systematic reviews, meta-analyses, and case reports were not considered.

Data extraction

A specific spreadsheet designated in Microsoft Excel was used for data extraction. For each included study, the following data were collected: 1) study-related data, including the last name of the first author, sample size, country of publication, and study design; 2) patients’ demographic data, including mean age, mean BMI, and gender distribution; 3) diagnostic approaches, including the used medications in DISE, the number of collapse sites detected (single- or multi-collapse), and other diagnostic modalities; 4) the levels of agreement between DISE and other approaches; and 5) post-therapeutic sleep parameters such as AHI and ESS.

Quality assessment

Quality assessment was performed using a specialized scale for non-randomized studies (the Newcastle-Ottawa quality assessment scale). Each study was assessed for three main domains, including comparability, selection, and outcomes and a total of nine items. The total Newcastle-Ottawa score (NOS) ranged between 0 (bad) and (9) excellent and a high-quality study was considered at NOS of ≥6.

Statistical analysis

The percentage of the change in therapeutic decisions was calculated as follows: the frequency of changed decisions/total number of patients *100. The percentage of change per site of collapse was depicted using Microsoft Excel and interpreted as added, reduced, or non-changed therapeutic decisions. Standard errors (SEs) were computed using the following formula: SE = sqrt [p(1-p)/n], where “p” indicates percentage and “n” indicates sample size. The pooled percentage change of all studies and the respective 95% confidence interval (CI) were calculated by entering the percentage change of every single study as well as the SE into RevMan software (v 5.3, Cochrane Collaboration). An *I*^2^ test was used for assessing statistical heterogeneity between studies, and a random-effects model was applied when there was significant heterogeneity. In the instance of significant heterogeneity, subgroup analysis was performed based on patients’ age (children or adults), sample size (<100 or >100), the applied DISE protocol, and the originally used diagnostic modality. Statistical significance was considered at *P *< 0.05.

## Results

Results of the search process

**Figure 1:**
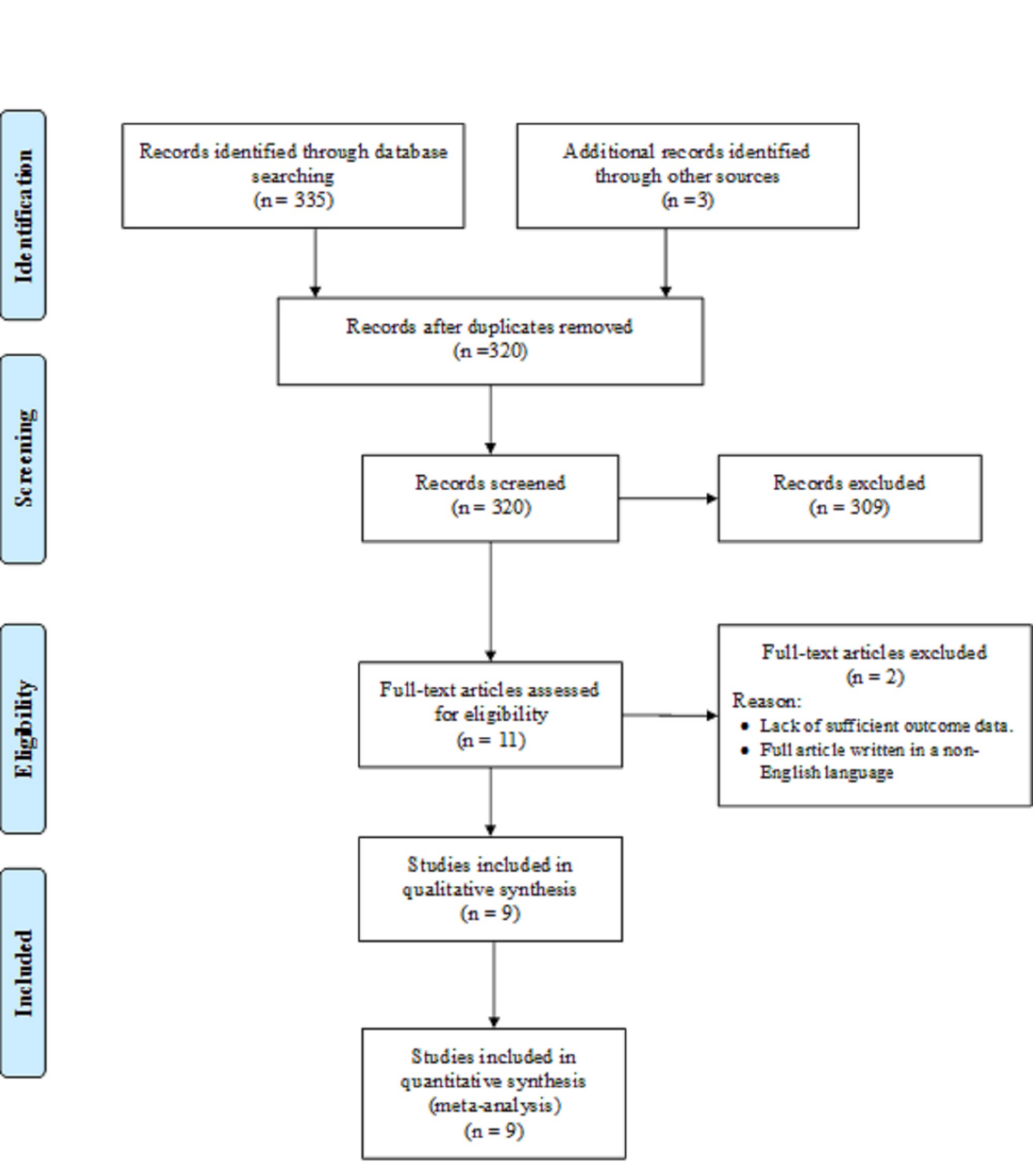
A PRISMA flow diagram showing the search process used in the present study PRISMA, Preferred Reporting Items for Systematic Reviews and Meta-analyses

Initially, 335 records were obtained from different databases, of which 18 duplicate records were identified and removed. Three records were identified by Google search. Thus, a total of 320 titles/abstracts were screened. The full-text version of 11 articles was assessed for inclusion. However, two studies were excluded, one that lacked a report on the primary outcome and the other being written in German. Ultimately, nine studies met the eligibility criteria (Figure 1).

Characteristics of the included studies

The included studies were published between 2009 and 2018. One study was conducted in the United States, another in Canada, and the remaining in European countries. One study employed a retrospective analysis of patients’ records to investigate the outcomes and another employed a case-control study, while the remaining studies were prospective cohort investigations [9-11]. Sample sizes ranged between 37 and 558. A total of 1247 patients were included (69.21% males). Children were recruited in three studies [10-12].

DISE-related findings

**Figure 2:**
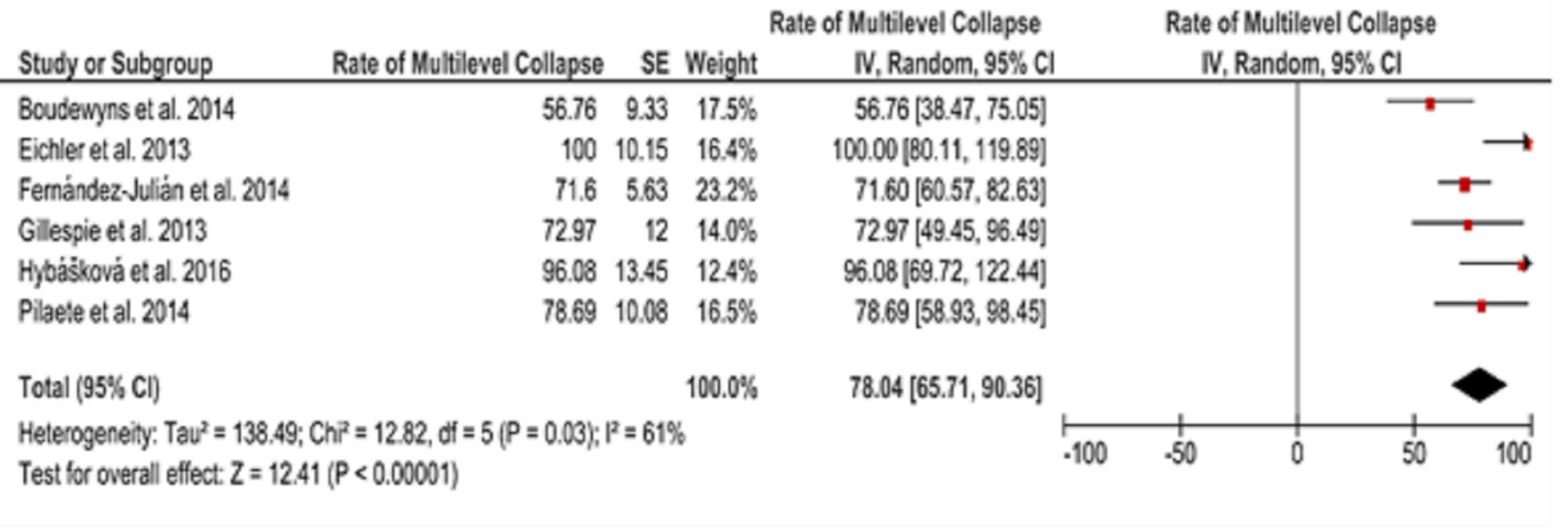
Forest plot depicting the rate of multilevel collapse in patients recruited in the included studies SE, standard error; CI, confidence interval; IV, interval variable

A combination of midazolam and propofol was used in the DISE protocols in three investigations (all were performed on adult patients) and midazolam was used only in one study, whereas propofol was solely administered in five studies [9-17]. In a total of six studies (where data was available), a multilevel collapse that contributed to OSA was detected in 78.04% of patients (CI, 65.71 to 90.36, Figure 2).

DISE changed the therapeutic decisions that were initially taken based on the airway endoscopy, the guidelines of the American Academy of Pediatrics (AAP), CBE only, or the findings of CBE, lateral cephalometry, and Müller maneuver (Table 1) [9-17].

**Table 1:** Characteristics of the included studies BMI, body mass index; DISE, drug-induced sleep endoscopy; F, female; M: male; NOS, the Newcastle-Ottawa score; T, total; TCI, target-controlled fusion [9-17]

Authors & Year	Country	Study Design	Mean age	Mean BMI (kg/m2)	M/F/T	Single/Multi-level collapse	Medication(s) used in DISE	NOS
Gillespie et al. 2013	USA	Prospective	46.3 ± 11.24	33.0 ± 7.25	21/16/37	10/27	Midazolam (0.7±0.7 mg) & glycopyrrolate (0.4±0.3 mg) during CBE, then propofol (192±59 mg) during DISE	6
Gazzaz et al. 2017	Canada	Retrospective	6.2 ± 2.7	NA	327/231/558	NA	Remifentanil (2-2.5 mcg/ml) & propofol (200-350 mcg/kg/min)	6
Collu et al. 2018	Italy	Case-control	4.67 ± 1.99	NA	98/52/150	NA	Sevoflurane for induction followed by propofol	6
Boudewyns et al. 2014	Belgium	Prospective	4.07 ± 0.9	15.7 ± 0.19	10/27/37	16/21	Propofol (1-2 mg initial bolus, then 6-10 mg/kg/h continuous infusion)	6
Pilaete et al. 2014	Belgium	Prospective	46.0 ± 11.41	28.67 ± 4.38	47/14/61	13/48	Initially, 1 mg midazolam in bolus, then propofol by TCI	6
Hewitt et al. 2009	UK	Prospective	45.5 ± 11.1	28.5 ± 4.6	77/17/94	NA	Midazolam (0.05 mg/kg) & propofol (1.5 mg/kg)	7
Eichler et al. 2013	Germany	Prospective	50.3 ± 11.60	27.4 ± 3.4	89/8/97	0/97	Midazolam (0.03 mg/kg initially, then increased by 1 mg until reaching a maximum of 0.2 mg/kg)	6
Hybášková et al. 2016	Czech Republic	Prospective	44.7 ± 11.3	30.1 ± 4.0	47/4/51	2/49	Propofol (1 mg/kg initially, then boluses of 10-20 mg every 3-5 minutes)	7
Fernández-Julián et al. 2014	Spain	Prospective	46.3 ± 6.4	28.1 ± 3.2	147/15/162	46/116	Propofol (via TCI)	7

Changes in therapeutic decisions

**Figure 3:**
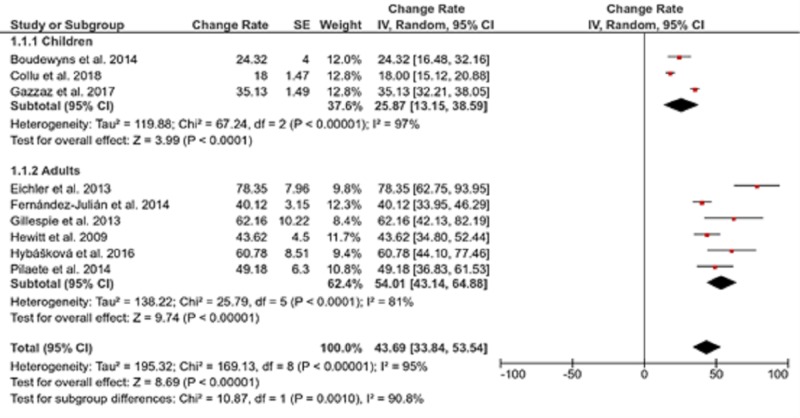
Forest plot showing the pooled change rate in therapeutic decisions after DISE DISE, drug-induced sleep endoscopy; SE, standard error; IV, interval variable; CI, confidence interval

DISE changed the recommended treatments in 43.69% of patients (CI, 33.84 to 53.54) and there was significant statistical heterogeneity between studies (*I*^2^ = 95%). The therapeutic decisions in adults changed more frequently (54.01%) than those in children (25.87%) and the difference was statistically significant (*P* = 0.001, Figure 3).

Subgroup analysis revealed that studies using propofol exclusively contributed to the reported heterogeneity, while there was no impact on patients’ age or sample size on reducing heterogeneity rates (Table 2 and Figure 3).

**Table 2:** Subgroup analysis of DISE-directed changes in therapeutic decisions based on sample size, the used DISE protocol, and change rates versus other diagnostic modalities AE, awake endoscopy; CBE, clinical basic examination (ENT); DISE, drug-induced sleep endoscopy; LC, lateral cephalometry; MM, Müller maneuver; AAP, The American Academy of Pediatrics; R, Random; F, fixed

Parameter	Studies	Model (I2%)	Change Rate (95% CI)	P
Sample size				
<100	6	R (90)	52.07 (36.26, 67.87)	0.05
>100	3	R (98)	30.92 (17.39, 44.45)	
DISE protocol				
Propofol	5	R (96)	33.99 (23.09, 44.89)	<0.001
Midazolam	1	NA	78.35 (62.75, 93.95)	
Propofol and Midazolam	3	F (30)	48.45 (39.76, 57.13)	
DISE versus other modalities				
CBE	6	R (96)	44.63 (27.89, 61.36)	0.02
AE	1	NA	62.16 (42.13, 82.19)	
CBE, LC, and MM	1	NA	40.12 (33.95, 46.29)	
AAP guidelines	1	NA	35.13 (32.21, 38.05)	

Notably, midazolam-based DISE techniques led to a significantly higher rate of change in therapeutic decisions (78.35%) as compared to a combination of midazolam and propofol (48.45%) and propofol exclusively (33.99%, *P* < 0.001). Furthermore, the change rates were significantly higher versus awake endoscopy (62.16%) as compared to CBE alone (44.63%), CBE plus lateral cephalometry and Müller maneuver (40.12%), and the AAP guidelines (35.13%, *P* = 0.02, Table 2).

**Figure 4:**
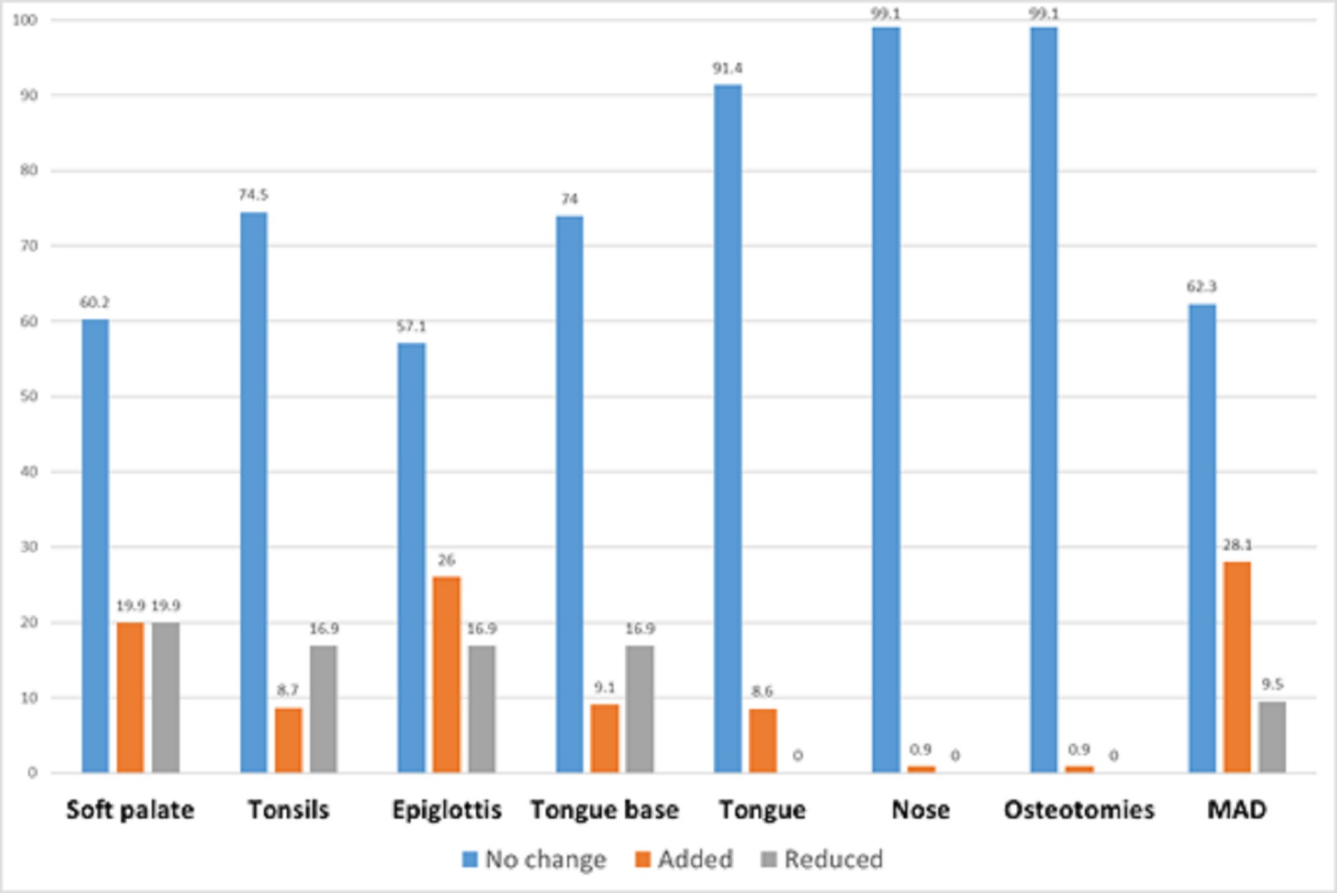
Surgical recommendations of DISE as compared to other diagnostic modalities in adults (n = 231) DISE, drug-induced sleep endoscopy; MAD, mandibular advancement devices

In adults, detailed descriptions of changes in surgical sites were provided in five studies among 231 patients [9,13-16]. The changed recommendations by DISE were frequent in the epiglottis (42.9%) and the soft palate (39.8%). Mandibular advancement devices (MADs) represented the most frequently added intervention after DISE (28.1%), followed by surgeries of the epiglottis (26%) and the soft palate (19.9%). Additionally, soft palate surgeries were the most commonly reduced operations (19.9%), followed by surgical indications of the tonsils, epiglottis, and tongue base (16.9% for each, Figure 4).

**Figure 5:**
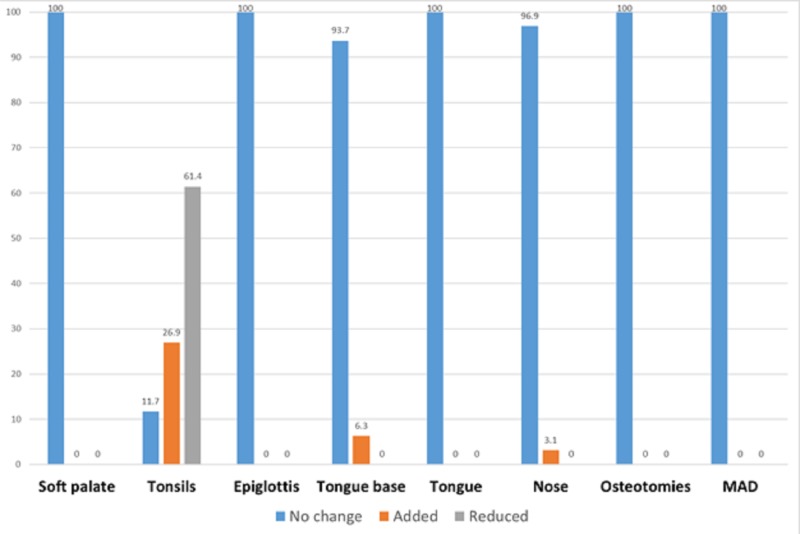
Surgical recommendations of DISE as compared to other diagnostic modalities in children (n = 223) DISE, drug-induced sleep endoscopy; MAD, mandibular advancement devices

In children, two studies revealed the changed sites of airway collapse [10-11]. Tonsillectomy was the most frequently changed procedure, with an 88.3% change rate. Tongue base and nose surgeries were also changed but far less frequently (6.3% and 3.1%, respectively, Figure 5).

Post-therapeutic outcomes after DISE recommendations

Eichler et al. found no significant correlation between changes in surgical plans as recommended by DISE and AHI. In a case-control study, Collu et al. also found no significant differences in the frequency of patients with an AHI of >10 per hour sleep postoperatively in patients whom surgeries were guided by DISE as compared to CBE-guided interventions. In the remaining studies, post-surgical outcomes were not reported or were not compared to preoperative values [9-11,13-17].

## Discussion

The identification of obstruction sites in OSA patients is of paramount importance for guiding subsequent therapeutic approaches. During sleep surgery, proper selection of sites of upper airway collapse might contribute to eventual surgical success for an individual patient. The role of DISE in directing surgical and non-surgical was investigated in the present study. We showed that DISE-directed decisions were altered in 43.69% of patients. Besides, approximately half of adult patients would have their therapeutic decisions changed, and the most frequent sites of changed therapies (added or reduced) were at the uvular and palatal levels. In the pediatric population, the changes were less apparent (in about one-quarter of them), while these changes were mostly exhibited at the tonsillar level.

It was not surprising that uvular and palatal sites were mostly changed as the treatment recommendations were altered concerning UPPP and RFP procedures in adults in most studies. Isolated palatal obstructions in OSA patients are frequent, where the soft palate is involved in 12.6% to 63.3% of cases [18]. The epiglottis is involved more commonly in multilevel collapse entities than in isolation [15]. There is a significant association between the presence of obstruction at the soft palate and less commonly, the epiglottis, and increased AHI and BMI [19]. Such an observation could be corroborated in our analysis, where there was a high rate of multilevel obstruction detected by DISE (80.5%) and the mean BMI ranged between 28.1 to 33.0 kg/m^2^. Even in patients with mild symptoms (AHI<5), DISE has a high sensitivity of detecting multilevel obstruction sites [20]. Multilevel surgeries require the precise identification of obstruction sites as well as a definitive assessment of the degree of collapse. Gillespie et al. stated that DISE had the ability to determine the level of surgical invasiveness by providing real-time pictures of collapse levels as well as the severity of collapse at each site [9].

These outcomes indicate the promising role of DISE in the identification of obstruction sites and hence affecting surgical decisions. Preoperative DISE can be performed rapidly within a mean time of nine minutes [9]. Additionally, DISE is indicated in patients with different OSA severity who failed or refused CPAP treatment. Furthermore, consistent with the progressively increased interest in non-CPAP treatments for OSA patients, it is important to improve prospective ways that predict the outcomes of treatment. Multiple studies have indicated the validity and interrater reliability of DISE [21-22]. It has been shown that two of three investigators reported significant variations in the patterns and degrees of collapse between DISE and awake intubation and that higher DISE scores were significantly associated with higher AHI [9].

As demonstrated in the present study, the changes implied by DISE could be significantly higher as compared to evaluation techniques performed during wakefulness. This is because awake techniques did not simulate the actual dynamics of collapsible upper airway structures during sleep. However, DISE findings can be further validated by comparing the DISE findings in OSA patients with those in normal subjects to directly emphasize the role of sedating agents on airway patency. This way, clinicians would be able to differentiate the reasons for airway obstruction, whether to be caused by their disease or anesthesia. Even if DISE is compared to upper airway investigations performed during natural sleep, the DISE technique is a time and manpower saving approach. Park et al. have recently compared the levels of agreement between a midazolam-based DISE approach and natural sleep endoscopy and found significant agreements regarding obstructions at the epiglottis (92.3%), the oropharynx lateral wall (88.5%), and the velum (76.9%) [23].

Safety during DISE procedures is another advantageous aspect. In general, no particular side effects have been reported. Central apnea has been occasionally demonstrated with rapid induction [24]. Certain investigators implemented oxygen saturation monitoring or applied a laryngeal mask or CPAP to manage deep desaturations [25]. Midazolam administration was also avoided in patients with severe OSA or high BMI [26]. Finally, propofol-driven impairment of the swallowing reflex might contribute to the accumulation of saliva, which hinders the direct visualization of obstruction sites in certain conditions [25]. However, it seems that the promising safety of DISE is directly related to propofol use. Such a medication induces a rapid anesthetic effect while preserving adequate spontaneous ventilation. The TCI system ensures a pharmacokinetic simulation pattern that prevents the loss of upper airway neuromuscular tone. Moreover, sedation depth could be controlled using a bispectral index (BIS) monitor [27].

On the other hand, the use of DISE has been subject to several challenges. First, there is currently no consensus about the methods used for sedation. The use of sedatives (either single or combined) may influence a muscular tone, leading to overestimating the severity of the collapse. Nonetheless, as previously mentioned, adequate application of TCI protocols might overcome such a problem. Second, a uniform classification system of obstruction sites has not been established. Multiple institutions have reported their own classification criteria, yet there is no definitive agreement on the ideal system [28]. Dijemeni et al. have recently found 14 DISE classification systems; all of them were based on non-randomized studies. In essence, most systems relied heavily on the identification of pharyngeal structures that contribute to obstruction to tailor individual treatment plans [29]. However, the authors underscored the subjective nature of DISE, creating a substantial divergence in the proposed treatment planning and therapeutic outcomes [29]. Third, sleep position may affect the visualization of obstruction sites, such that the collapse would be significantly improved in the lateral sleep position. As such, DISE should be performed in multiple sleep positions to approach the best therapeutic decision. Finally, DISE performance can be affected by the physical condition of the patient prior to the procedure. Park and Kim investigated DISE findings after a treadmill stress test and compared the outcomes to another control group without exercise [30]. The authors found significant changes in obstruction levels at the velum, oropharynx, and tongue base between groups, causing either under or overestimation of airway obstruction.

We encountered multiple limitations in the present study. The significant heterogeneity was consistent among all studies except for those employing a combination of midazolam and propofol [9,13-14]. Future investigations with large sample sizes, prospective designs and, possibly, using a unified DISE classification system might contribute to reducing the observed heterogeneity. Another limitation is the lack of reporting of the outcomes related to sleep parameters. The inclusion of comparative studies, in which two or more groups were allocated to DISE and other diagnostic modalities, was not possible since these studies would not reveal changes in surgical recommendations by DISE. Therefore, important data about the postoperative outcomes of DISE were not considered.

## Conclusions

The DISE approach has been found to play a major role in changing treatment decisions, with approximately half of the OSA patients altering their treatment approaches. Changed surgeries involved the epiglottis and the soft palate more frequently than other sites of obstruction, while the rates of change were significantly higher in adults than children and when using midazolam-based DISE as compared to other protocols. Besides, DISE-directed surgeries were different from those recommended by awake endoscopy at a significantly higher rate than CBE alone or CBE with other modalities. A unified classification system regarding obstruction sites revealed by DISE is needed. Additionally, future studies should investigate treatment outcomes, safety, and reliability of DISE-driven procedures.
